# Value of repeated imaging in patients with a stroke who are transferred for endovascular treatment

**DOI:** 10.1136/neurintsurg-2020-017050

**Published:** 2021-03-08

**Authors:** Laura C C van Meenen, Nerea Arrarte Terreros, Adrien E Groot, Manon Kappelhof, Ludo F M Beenen, Henk A Marquering, Bart J Emmer, Yvo B W E M Roos, Charles B L M Majoie, Jonathan M Coutinho

**Affiliations:** 1 Department of Neurology, Amsterdam UMC, University of Amsterdam, Amsterdam, North Holland, The Netherlands; 2 Department of Biomedical Engineering and Physics, Amsterdam UMC, University of Amsterdam, Amsterdam, North Holland, The Netherlands; 3 Department of Radiology and Nuclear Medicine, Amsterdam UMC, University of Amsterdam, Amsterdam, North Holland, The Netherlands

**Keywords:** stroke, thrombectomy, CT

## Abstract

**Background:**

Patients with a stroke who are transferred to a comprehensive stroke center for endovascular treatment (EVT) often undergo repeated neuroimaging prior to EVT.

**Objective:**

To evaluate the yield of repeating imaging and its effect on treatment times.

**Methods:**

We included adult patients with a large vessel occlusion (LVO) stroke who were referred to our hospital for EVT by primary stroke centers (2016–2019). We excluded patients who underwent repeated imaging because primary imaging was unavailable, incomplete, or of insufficient quality. Outcomes included treatment times and repeated imaging findings.

**Results:**

Of 677 transferred LVO stroke, 551 were included. Imaging was repeated in 165/551 patients (30%), mostly because of clinical improvement (86/165 (52%)) or deterioration (40/165 (24%)). Patients who underwent repeated imaging had higher door-to-groin-times than patients without repeated imaging (median 43 vs 27 min, adjusted time difference: 20 min, 95% CI 15 to 25). Among patients who underwent repeated imaging because of clinical improvement, the LVO had resolved in 50/86 (58%). In patients with clinical deterioration, repeated imaging led to refrainment from EVT in 3/40 (8%). No symptomatic intracranial hemorrhages (sICH) were identified. Ultimately, 75/165 (45%) of patients with repeated imaging underwent EVT compared with 326/386 (84%) of patients without repeated imaging (p<0.01).

**Conclusions:**

Neuroimaging was repeated in 30% of patients with an LVO stroke and resulted in a median treatment delay of 20 minutes. In patients with clinical deterioration, no sICH were detected and repeated imaging rarely changed the indication for EVT. However, in more than half of patients with clinical improvement, the LVO had resolved, resulting in refrainment from EVT.

## Introduction

Endovascular treatment (EVT) is routine care for patients with anterior circulation large vessel occlusion (LVO) acute ischemic stroke.^1–3^ EVT can be performed only in specialized hospitals, so called comprehensive stroke centers (CSCs). In most countries, paramedics transport patients with a suspected stroke to the nearest hospital for diagnostic work-up and initiation of intravenous thrombolysis (IVT). Usually, this nearest hospital is not a CSC, but a primary stroke center (PSC). Thus, patients who are eligible for EVT must subsequently be transferred to a CSC.

On arrival at the CSC, neuroimaging is often repeated, although in varying frequencies.^4–8^ Repeated imaging may provide information on change in Alberta Stroke Program Early CT Score (ASPECTS),^6 9 10^ thrombus migration, recanalization, and intracranial hemorrhage after IVT. Such findings can be clinically relevant and may result in the decision to refrain from EVT. Repeating imaging may thus reduce the number of futile diagnostic angiographies, and the associated risks of these procedures, such as femoral artery dissections, thromboembolic complications, and anesthesia complications.^11^ In addition, avoiding unnecessary EVT procedures reduces healthcare costs. On the other hand, repeating imaging itself also adds healthcare costs and increases contrast medium exposure for patients. Moreover, performing additional imaging delays treatment, which can negatively affect the prognosis of patients.^12^ With our study, we aimed to evaluate the diagnostic yield and the treatment delay caused by repeating imaging in patients with an LVO stroke who are transferred from a PSC for EVT.

## Materials and methods

Data will not be made available to other researchers, as no patient approval was obtained for sharing coded data. However, syntax and output files of statistical analyses can be made available on request.

### Study design and population

We performed a single-center cohort study, using data from our prospective stroke registry. Our hospital receives EVT referrals from 11 nearby PSCs and has a catchment area for EVT of approximately 3.3 million inhabitants. We included adult patients with acute ischemic stroke due to an LVO, who were primarily presented to a PSC and subsequently referred to our hospital for EVT. We used data of patients referred between January 2016 and June 2019. We excluded patients who underwent repeated imaging because imaging from the referring hospital was unavailable, of insufficient quality, or incomplete, including the need to perform CT perfusion in patients who presented more than 6 hours after symptom onset. The procedures followed were all in accordance with institutional guidelines. All patients eligible for inclusion were sent a letter with detailed information about the study. The patient or legal representative had the opportunity to deny permission for use of their data via an opt-out form, in accordance with the European Union General Data Protection Regulation.

### Definitions, procedures, and outcomes

EVT was defined as arterial puncture in the angiography suite, with the aim of performing mechanical thrombectomy with a stent retriever and/or thrombus aspiration. The exact EVT strategy was at the discretion of the interventionist. Time of stroke onset was defined as the time of witnessed symptom onset or, if this was unknown, the time that the patient was last known to be well. All imaging was assessed as part of standard clinical practice.

The National Institutes of Health Stroke Scale (NIHSS) was used to quantify the severity of neurological deficit. If no NIHSS score was reported by the treating physician, it was scored retrospectively, as previously published.^13^

Our primary outcome was time from arrival at the CSC to groin puncture (CSC DTGT). Other workflow-related outcomes were time from stroke onset to groin puncture (OTGT) and time from arrival at the PSC to groin puncture (PSC DTGT). Clinical outcome measures were good functional outcome at 90 days post-stroke, defined as a score of 0–2 on the modified Rankin Scale (mRS), overall shift in mRS score between groups, occurrence of symptomatic intracranial hemorrhage (sICH), and mortality at 90 days post-stroke. Repeated imaging findings were scored separately for non-contrast CT (NCCT) and CT angiography (CTA). On NCCT, the presence or absence of intracranial hemorrhage (ICH) was scored. ICH was defined as symptomatic if the patient died or deteriorated neurologically (an increase of ≥4 points on the NIHSS) as a result of the hemorrhage.^14^ On CTA, the presence or absence of an LVO was scored, and its location was compared with PSC imaging (vascular territory and segment). LVO was defined as an occlusion of the intracranial part of the internal carotid artery (ICA), the first segment of the middle cerebral artery (M1), the proximal part of the second segment (after first bifurcation) of the middle cerebral artery (proximal M2), the first segment of the anterior cerebral artery, or the basilar artery. A persistent LVO was defined as an LVO in the same vascular territory on repeated imaging, even if the vascular segment had changed.

### Statistical analysis

We compared patients in whom neuroimaging (NCCT and/or CTA) was repeated on arrival at the CSC with patients who did not undergo repeated imaging. Baseline characteristics were compared using independent samples t-test for normally distributed continuous variables, Mann-Whitney U test for non-normally distributed continuous variables, and χ^2^ test for categorical variables. Multivariable linear regression was used for the analyses of treatment times. The analysis of CSC DTGT was adjusted for the following potential confounders (unless reported otherwise, baseline characteristics were measured on arrival at the CSC): age, previous stroke, NIHSS score, location of occlusion on first CTA scan, and presentation outside office hours. For the analyses of OTGT and PSC DTGT, we adjusted for age, previous stroke, NIHSS score, location of occlusion on first CTA scan, presentation outside office hours, and treatment with IVT. Binary logistic regression was used for the analyses of good functional outcome, sICH, and mortality. Ordinal logistic regression was used to assess the overall shift in mRS score between groups. These regression analyses were adjusted for age, blood pressure, previous stroke, NIHSS score, location of occlusion on first CTA scan, time of presentation (within or outside office hours), treatment with IVT, and endovascular treatment (EVT). For all regression analyses, we imputed missing data using multiple imputation for variables with more than 10% of missing values, using the following covariates: age, sex, previous stroke, diabetes, atrial fibrillation, coronary artery disease, blood pressure, baseline NIHSS score, location of occlusion, treatment with IVT, EVT, OTGT, PSC DTGT, expanded Treatment In Cerebral Ischemia score after EVT and 90-day mRS score. Analyses were performed using SPSS (version 25; SPSS Inc, Chicago, Illinois, USA).

## Results

**Figure 1:**
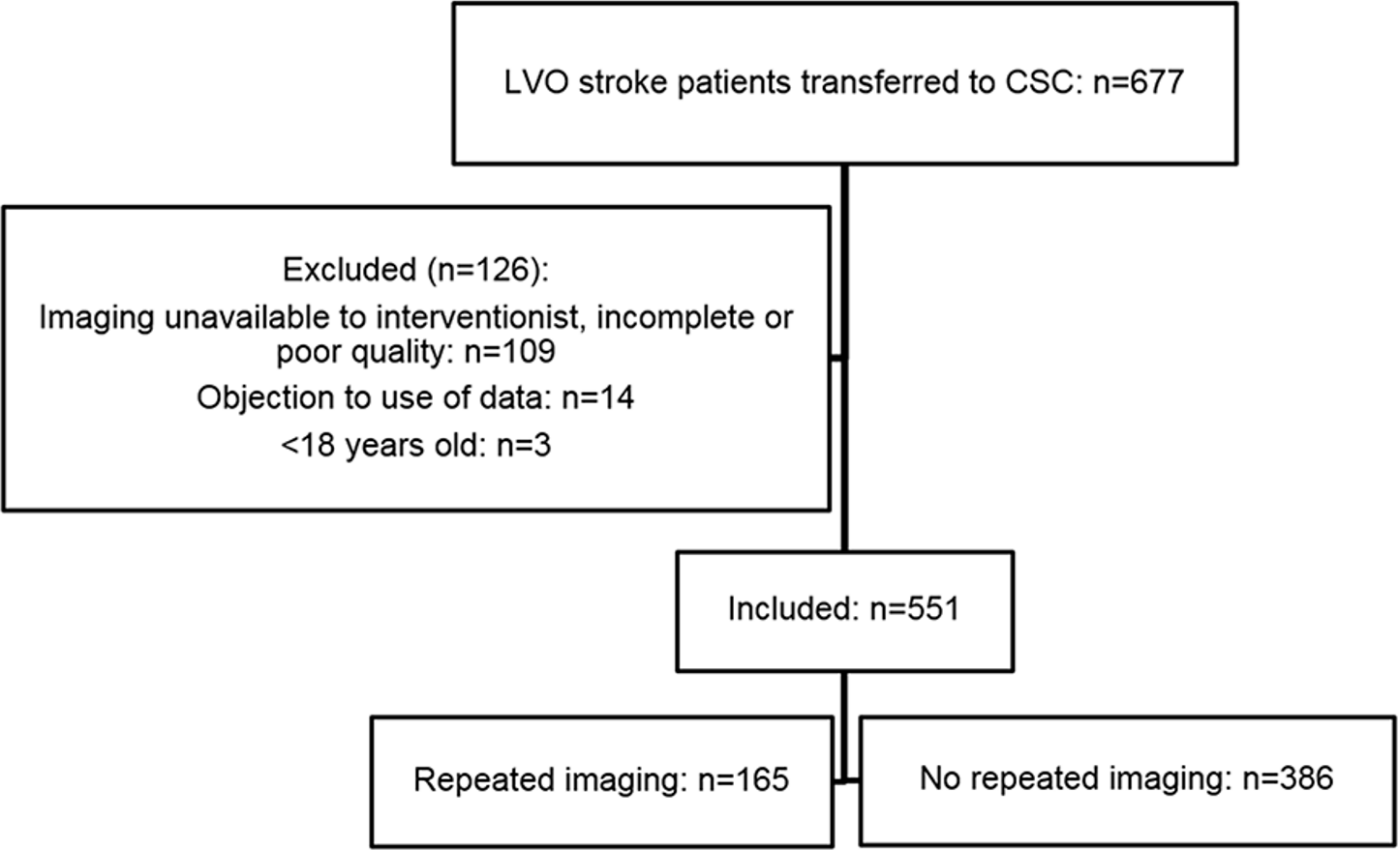
Flow chart of patient inclusion. CSC=comprehensive stroke center; LVO=large vessel occlusion.

Within the study period, 677 patients with LVO stroke were transferred from one of the PSCs to our hospital for EVT. Of these, 126 were excluded for the following reasons: primary imaging unavailable, incomplete or of poor quality (n=109); objection to use of data (n=14); and age <18 years (n=3). Therefore, we included 551 patients in the current analysis (figure 1). Repeated imaging was performed in 165/551 (30%) of these patients. The change in proportion of patients who underwent repeated imaging over time is depicted in online supplemental figure 2. The most common reasons for repeating imaging were clinical improvement (86/165 (52%)) and clinical deterioration (40/165 (24%)). Other reasons are reported in table 1.

10.1136/neurintsurg-2020-017050.supp1 Supplementary data

**Table 1:** Baseline characteristics

Characteristics*	Repeated imaging (n=165)	No repeated imaging (n=386)	*p* value
Age, years – mean±SD	71±15.3	70±13.3	0.40
Male sex – no./total (%)	79/165 (48%)	200/386 (52%)	0.40
Hypertension – no./total (%)	68/164 (41%)	150/382 (39%)	0.63
Diabetes mellitus – no./total (%)	21/164 (13%)	56/382 (15%)	0.57
Atrial fibrillation – no./total (%)	34/164 (21%)	95/382 (25%)	0.30
Coronary artery disease – no./total (%)	14/164 (9%)	80/382 (21%)	<0.01
Previous stroke – no./total (%)	30/164 (18%)	66/382 (17%)	0.78
Pre-stroke mRS score† – median (IQR)	1 (0–1)	1 (0–1)	0.72
Systolic blood pressure (mm Hg) – mean±SD	150±28.1	148±23.9	0.37
Diastolic blood pressure (mm Hg) – mean±SD	83±17.0	82±16.5	0.65
NIHSS score at PSC arrival‡ – median (IQR)	11 (7–15)	13 (9–17)	<0.01
NIHSS score at CSC arrival§ – median (IQR)	9 (4–17)	16 (10–20)	<0.01
Intracranial occlusion site (on PSC imaging) – no./total (%)			0.07
ICA	26/164 (16%)	76/386 (20%)	
M1	90/164 (55%)	228/386 (59%)	
Proximal M2	31/164 (19%)	38/386 (10%)	
Anterior cerebral artery	0/164 (0%)	1/386 (0%)	
Basilar artery	13/164 (8%)	31/386 (8%)	
No LVO (misread by radiologist at PSC)	4/164 (2%)	12/386 (3%)	
Reason for repeating imaging – no./total (%)			
Clinical improvement	86/165 (52%)	NA	–
Clinical deterioration	40/165 (24%)	NA	–
Additional imaging characteristics for assessing indication for EVT**	11/165 (7%)	NA	–
Other	6/165 (4%)	NA	–
Reason not recorded	22/165 (13%)	NA	–
Presentation outside office hours – no./total (%)	74/150 (49%)	233/365 (64%)	<0.01
Time from stroke onset to arrival at PSC (min)¶ – median (IQR)	50 (30–81)	58 (32–95)	0.14
Treatment with IVT – no./total (%)	135/165 (82%)	276/386 (72%)	0.01

*All baseline characteristics were measured on arrival at the CSC, unless reported otherwise.

†Number of missing values: 350.

‡28.

§4.

¶178.

**For example, ASPECTS, collaterals, core/penumbra ratio <6 hours.

ASPECTS, Alberta Stroke Program Early CT Score; CSC, comprehensive stroke center; EVT, endovascular treatment; ICA, intracranial part of internal carotid artery; IVT, intravenous thrombolysis; LVO, large vessel occlusion; M1, first segment of the middle cerebral artery; M2, proximal part of the second segment (after first bifurcation) of the middle cerebral artery; mRS, modified Rankin Scale; NA, not applicable; NIHSS, National Institutes of Health Stroke Scale; no., number; PSC, primary stroke center.

Baseline characteristics for patients with and without repeated imaging are shown in table 1. In the repeated imaging group, patients more often had received IVT (82% vs 72%, p=0.01) and presentation outside office hours was less common (49% vs 64%, p<0.01). Coronary artery disease was less prevalent in the repeated imaging group (9% vs 21%, p<0.01). There were slightly fewer ICA and M1 occlusions and slightly more proximal M2 occlusions in the repeated imaging group, although this difference was not statistically significant (table 1). Patients who underwent repeated imaging because of clinical improvement (86/165 (52%)), had a median change in NIHSS score of −5 between PSC and CSC (IQR −8 to −2). In patients with clinical deterioration (40/165 (24%)), NIHSS scores had increased by a median of 6 points on arrival at the CSC (IQR 3 to 9).

Patients who underwent repeated imaging had longer CSC DTGT (median 43 vs 27 min, adjusted time difference: 20 min, 95% CI 15 to 25) and PSC DTGT (median 147 vs 124 min, adjusted time difference: 27 min, 95% CI 14 to 40). The OTGT did not differ between groups (table 2). The odds of good functional outcome at 90 days post-stroke (mRS score 0–2) were higher for the repeated imaging group, but this association dissipated after adjusting for potential confounders (unadjusted OR 1.57, 95% CI 1.01 to 2.44; adjusted OR 1.10, 95% CI 0.61 to 1.99; online supplemental table I. Symptomatic ICH was numerically less frequent in the repeated imaging group, but this was not statistically significant after adjustment (1% vs 8%; unadjusted OR 0.15, 95% CI 0.03 to 0.62; adjusted OR 0.29, 95% CI 0.07 to 1.31). Other clinical outcomes did not differ between groups (online supplemental table I).

**Table 2:** EVT-related outcomes

	Repeated imaging (n=165)	No repeated imaging (n=386)	P value	
Groin puncture – no./total (%)	75/165 (45%)	326/386 (84%)	<0.01	
Persistent LVO, ≥1 MT attempt(s)	57/165 (35%)	261/386 (68%)	<0.01	
Persistent LVO, no access to occlusion location	12/165 (7%)	35/386 (9%)	0.49	
LVO resolved, angiography only*	6/165 (4%)	29/386 (8%)	0.09	
**Subgroup: patients who underwent groin puncture**				
	**Repeated imaging (n=75**)	**No repeated imaging (n=326**)	**Unadjusted β (95% CI**)	**Adjusted β (95% CI**)
CSC door-to-groin time§ – median (IQR)	43 (35 to 59)	27 (19 to 37)	19.9 (14.7 to 25.1)	20.0 (14.8 to 25.3)†
PSC door-to-groin time¶ – median (IQR)	147 (118 to 190)	124 (104 to 154)	22.6 (9.6 to 35.6)	26.9 (14.2 to 39.6)‡
Onset-to-groin time** – median (IQR)	198 (167 to 261)	195 (156 to 249)	11.1 (-9.6 to 31.9)	15.0 (-6.0 to 35.9)‡

*Among patients who underwent a cerebral angiography only, one periprocedural complication was reported (femoral pseudoaneurysm, in the repeated imaging group).

†Adjusted for: age, previous stroke, NIHSS score, location of occlusion on first CTA, presentation outside office hours.

‡Adjusted for: age, previous stroke, NIHSS score, location of occlusion on first CTA, presentation outside office hours, treatment with intravenous thrombolysis.

§Number of missing values: 27.

¶55.

**17.

CSC, comprehensive stroke center; CTA, CT angiography; EVT, endovascular treatment; LVO, large vessel occlusion; MT, mechanical thrombectomy; NIHSS, National Institutes of Health Stroke Scale; no., number; PSC, primary stroke center.

Among patients with repeated imaging, NCCT was redone at the CSC in 73%, and CTA in 75% of patients (both in 48%). The diagnostic yield of repeated imaging is shown in table 3. Among all patients with a repeated NCCT, only one ICH was found, which was asymptomatic and did not occur in a patient with clinical deterioration. Of all patients with a repeated CTA, 67/124 (54%) had a persistent LVO, 11/67 (16%) of which had migrated to a more distal segment. One LVO was found in a new vascular territory. In 57/124 (46%) of patients, the LVO had resolved. In patients with clinical improvement—that is, with a decrease in NIHSS score, the LVO had resolved more often on repeated imaging (p<0.01; please see online supplemental figure 3). When analyzed separately, in patients who underwent repeated CTA because of clinical improvement, the LVO had resolved in 50/86 (58%) of patients. In patients who underwent repeated CTA because of clinical deterioration, CTA showed that the LVO had resolved in 3/13 (23%) of patients (table 3). In the remaining 27 patients with clinical deterioration, CTA was not repeated.

**Table 3:** Repeated imaging findings

Imaging modality	All patients (n=165)	Clinical improvement* (n=86)	Clinical deterioration† (n=40)
NCCT‡			
Symptomatic ICH	0/120 (0%)	0/50 (0%)	0/38 (0%)
Asymptomatic ICH	1/120 (1%)	0/50 (0%)	0/38 (0%)
CTA§			
Persistent LVO	67/124 (54%)	36/86 (42%)	10/13 (77%)
Same segment	55/124 (44%)	28/86 (33%)	8/13 (62%)
Distal migration	11/124 (9%)	8/86 (9%)	2/13 (15%)
New vascular territory	1/124 (1%)	0/86 (0%)	0/13 (0%)
LVO resolved	57/124 (46%)	50/86 (58%)	3/13 (23%)

*Median Δ NIHSS score between PSC and CSC: −5 (−8 to −2).

†Median Δ NIHSS score between PSC and CSC: 6 (3 to 9).

‡NCCT was repeated in 120/165 patients (73%); 50/86 (58%) of patients with clinical improvement and 38/40 (95%) of patients with clinical deterioration.

§CTA was repeated in 124/165 patients (75%); all patients with clinical improvement and 13/40 (33%) of patients with clinical deterioration.

CSC, comprehensive stroke center; CTA, CT angiography; ICH, intracranial hemorrhage; LVO, large vessel occlusion; NCCT, non-contrast CT; PSC, primary stroke center.

Ultimately, 75/165 (45%) of the repeated imaging group underwent EVT, versus 326/386 (84%) of patients without repeated imaging. In the repeated imaging group, fewer patients underwent cerebral angiography only, although this difference was not statistically significant (4% vs 8%, p=0.09; table 2). Among patients who underwent angiography only (n=35), one periprocedural complication (2.9%) was reported, which occurred in the repeated imaging group. This complication was a femoral pseudoaneurysm, which was treated with an ultrasound-guided thrombin injection and resolved without sequelae. Reasons for refraining from groin puncture are reported for both groups in online supplemental table II. In 61/165 (37%) of patients who underwent repeated imaging, the findings on repeated imaging resulted in, or contributed to, the decision to refrain from EVT. This was the case for 49/86 (57%) of patients with clinical improvement and 3/40 (8%) of patients with clinical deterioration.

## Discussion

In this single-center cohort study of patients with an LVO stroke who were transferred for EVT, neuroimaging was repeated in 30% of patients on arrival at the CSC, resulting in a median treatment delay of 20 minutes. In patients with clinical deterioration, repeated imaging rarely resulted in the decision to refrain from EVT, and no sICH was detected. On the other hand, in more than half of patients with clinical improvement, the LVO had resolved, abolishing the need for EVT altogether.

Reports on the frequency with which imaging is repeated in patients transferred for EVT vary substantially. Venema *et al* reported that in CSCs in the Netherlands, on average, NCCT is repeated in 6% and CTA in 5% of transferred patients prior to EVT.^7^ However, that study excluded transferred patients in whom EVT was ultimately not performed, probably leading to an underestimation of the true frequency of repeating imaging in patients with an LVO stroke. Other studies report repeated imaging rates of up to 86% of patients, or even in all transferred patients as standard practice.^4–6 8^ Little has been reported about the diagnostic yield of repeated imaging in patients transferred for EVT or its effects on work flow. Several previous studies have found that presentation of patients directly to the angiography suite, instead of to the emergency room, substantially reduces DTGT.^15–17^ For instance, Jadhav *et al* found that in patients transferred for EVT, DTGT was reduced by 59 minutes when the emergency room was bypassed.^17^ The authors hypothesized that this was partly due to a reduction in repeating imaging. However, presenting patients directly to the angiography suite requires around-the-clock availability of an angiography suite and personnel, which is often not feasible and increases healthcare costs. Compared with previous literature, the percentage of patients who ultimately did not undergo EVT was relatively low in our population. Two previous studies have reported 41% and 45% of futile interhospital transfers,^4 8^ whereas in our study this was the case for 27% of patients. Potential explanations for this finding could be longer travel times or different selection methods for transferring patients for EVT.

We found several baseline imbalances that should be noted. First, NIHSS scores on arrival at the CSC were lower for the repeated imaging patients. Most likely, this is because clinical improvement was a common reason for CSC physicians to repeat imaging. Interestingly, NIHSS scores at the PSC were also lower for the repeated imaging group. This could be because the repeated imaging group contained slightly more patients with an M2 occlusion, which are associated with less severe neurological deficits than more proximally located LVOs, and which more often show early recanalization after IVT.^18^ Another possible explanation is that CSC physicians might have been more inclined to repeat imaging in patients with less severe neurological deficits, independent of the change in NIHSS score. Second, the percentage of patients who received IVT was higher in the repeated imaging group. It seems plausible that this is because IVT caused part of the LVOs to resolve, resulting in clinical improvement, which again was a common reason for CSC physicians to repeat imaging. Third, a history of coronary artery disease was less prevalent in the repeated imaging group. A possible explanation for this finding could be that patients with coronary artery disease, and thus atherosclerosis, more often have an atherosclerotic etiology of their stroke. Previous studies have reported that atherosclerotic stroke is more often refractory to IVT,^19 20^ which might have resulted in less clinical improvement and therefore less often repeated imaging.

Several limitations to our study warrant mentioning. First, imaging was not assessed by a core laboratory, but assessed only as part of standard clinical practice. Some imaging characteristics, such as ASPECTS, collateral score, and core/penumbra volumes on CT perfusion therefore were not systematically scored. These imaging characteristics might have influenced the decision as to whether or not to perform EVT. Although CT perfusion volumes are strictly only indicated for patients in the ‘late’ time window—who were excluded from our analyses if the need for obtaining CT perfusion imaging was the sole reason for repeating imaging—we cannot exclude the possibility that CT perfusion characteristics nonetheless affected treatment decisions. However, if these imaging characteristics were reported as a reason for refraining from EVT, we included this in our results. Second, it is important to note that this study took place in the Netherlands, which is a densely populated country, in which hospitals are located relatively close to one another and which has an overall good infrastructure. As a result, both transfer times and times between acquisition of primary imaging and repeated imaging were relatively short. Consequently, our findings should be extrapolated with caution to hospital systems with longer travel times between centers. Finally, for three variables, we had relatively high numbers of missing values: pre-stroke mRS score (64%), mRS score at 90 days post-stroke (32%), and time of patient arrival at the PSC (23%). We tried to minimize the impact of the missing data on our analyses by using multiple imputation.

Future research on this topic could focus on developing a prediction model for early recanalization, in order to help avoid futile interhospital transfer.

In conclusion, patients transferred to our CSC for EVT underwent repeated neuroimaging in 30% of cases. Repeating imaging delayed treatment by approximately 20 minutes. In patients with clinical deterioration, the yield of repeating imaging was low and no sICH prior to EVT were identified. In patients with clinical improvement, repeated imaging showed that the LVO had resolved in 58% of cases and thereby resulted in refrainment from EVT. Based on our findings, repeating neuroimaging does not seem beneficial in patients with clinical deterioration, but is very useful in patients with clinical improvement, since it helps avoid futile diagnostic angiographies in more than half of this population.

## Data Availability

Individual patient data cannot be made available under Dutch law because we did not obtain patient approval for sharing individual patient data, even in coded form. However, all syntax files and output of statistical analyses will be made available upon reasonable request.
