# Supplementary material for: Value of repeated imaging in patients with a stroke who are transferred for endovascular treatment
Source: J Neurointerv Surg. 2021 Mar 8;14(1):neurintsurg-2020-017050. doi: 10.1136/neurintsurg-2020-017050 (PMC8784993; doi:10.1136/neurintsurg-2020-017050)
Supplement: Supplementary data [file neurintsurg-2020-017050supp001.pdf]

## SUPPLEMENTAL MATERIAL

## Tables

### Table I. Clinical outcomes

| | Repeated imaging, n=165 | No repeated imaging, n=386 | Unadjusted OR (95% CI) | Adjusted OR‡ (95% CI) |
|---|---|---|---|---|
| **Functional independence (90 day mRS 0-2)** – no./total (%) | 45/95 (47%) | 113/279 (41%) | 1.57 (1.01 – 2.44) | 1.10 (0.61 – 1.99) |
| **mRS score at 90 days*** – median (IQR) | 3 (1-6) | 3 (1-6) | 1.55† (0.95 – 2.54) | 1.14† (0.70 – 1.88) |
| **Symptomatic ICH** – no./total (%) | 2/164 (1%) | 30/383 (8%) | 0.15 (0.03 – 0.62) | 0.29 (0.07 – 1.31) |
| **Mortality at 90 days** – no./total (%) | 31/95 (33%) | 78/279 (28%) | 0.94 (0.61 – 1.46) | 0.82 (0.45 – 1.50) |

CI = confidence interval; ICH = intracranial hemorrhage; IQR = interquartile range; mRS = modified Rankin scale; OR = odds ratio.

Number of missing values: *177.

†Odds of 1-point shift towards a favorable outcome on the mRS for the repeated imaging group.

‡All analyses were adjusted for: age, blood pressure, previous stroke, NIHSS score, location of occlusion on first CTA, time of presentation (within or outside office hours), treatment with IVT, treatment with EVT.

**Table II. Reasons for refraining from EVT**

| | **Repeated imaging (n=90)** | **No repeated imaging (n=60)** | ***p* value** |
|---|---|---|---|
| Reason for refraining from EVT | | | <0.01 |
| Clinical characteristics – no./total (%) | 21/90 (23%) | 37/60 (62%) | |
| Combination of clinical and radiological characteristics – no./total (%) | 6*/90 (7%) | 9/60 (15%) | |
| Radiological characteristics – no./total (%) | 63†/90 (70%) | 13/60 (22%) | |
| LVO resolved | 55/90 (61%) | 0/60 (0%) | |
| No LVO on PSC imaging‡ | 0/90 (0%) | 10/60 (17%) | |
| Unfavorable imaging characteristics§ | 3/90 (3%) | 2/60 (3%) | |
| EVT not technically feasible | 3/90 (3%) | 1/60 (2%) | |
| Other | 2/90 (2%) | 1/60 (2%) | |

ASPECTS = Alberta stroke program early CT score; EVT = endovascular treatment; LVO = large vessel occlusion; no. = number; PSC = primary stroke center.

*In 3/6 patients, repeated imaging contributed to the decision to refrain from EVT; in the other 3/6 patients, the imaging factors contributing to the decision to refrain from EVT were (also) visible on PSC imaging.

†In 58/63 patients, repeated imaging contributed to the decision to refrain from EVT; in the other 5/63 patients, the imaging factors contributing to the decision to refrain from EVT were (also) visible on PSC imaging.

‡Upon reassessment by the neuro-interventional radiologist at the CSC

§Low ASPECTS, poor collateral status and/or unfavorable CT perfusion characteristics.

**Figures**

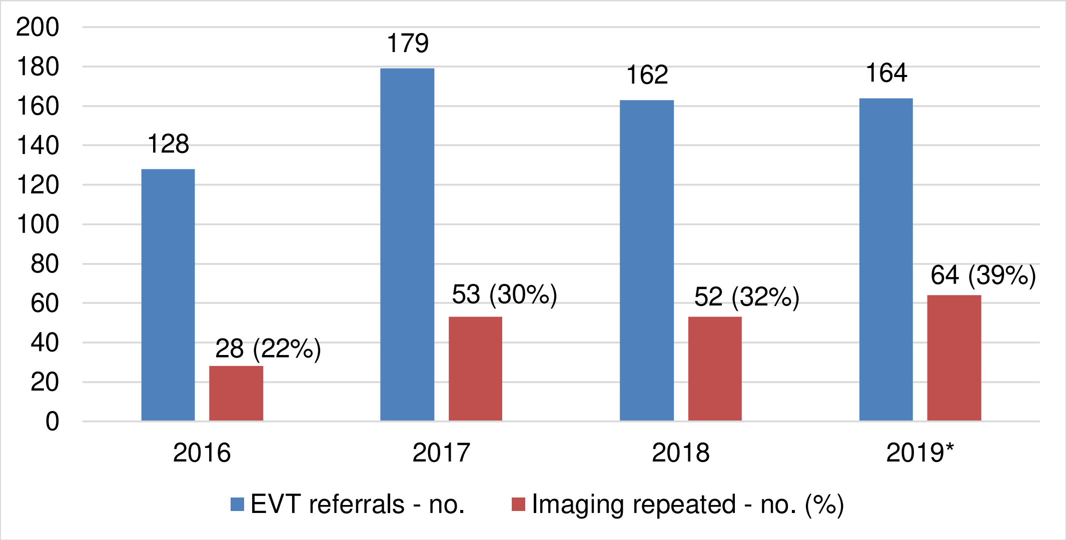


**Figure 2. Referrals for EVT per year during the study period.** Number of referrals (blue) and number and percentage of patients with repeated imaging (red) are reported for our hospital during the study period (January 2016 – June 2019). *For 2019, data were extrapolated for the remainder of the year. EVT = endovascular treatment; no. = number.

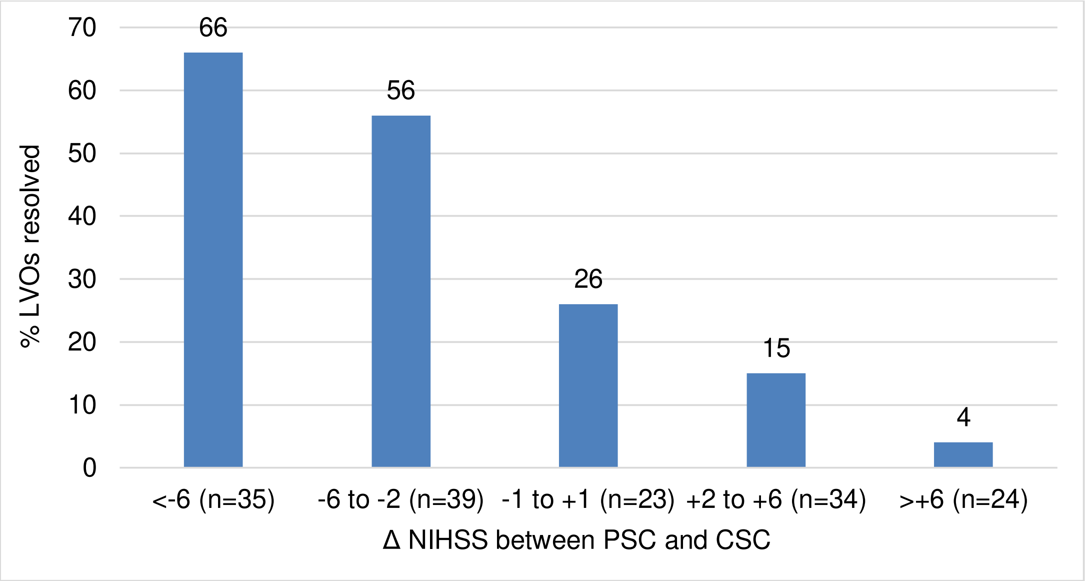


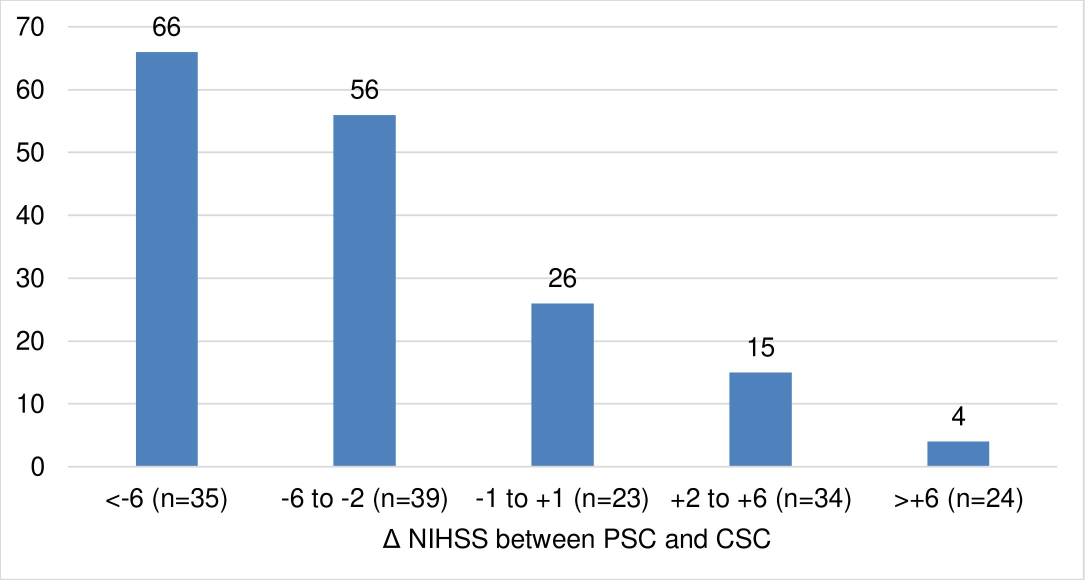


**Figure 3. Percentage of resolved LVOs per Δ NIHSS sub group.** The percentage of LVOs that were resolved on repeated CTA related to the change in NIHSS score between PSC and CSC. A negative Δ NIHSS value signifies clinical improvement; a positive value means clinical deterioration. The lower the Δ NIHSS, the higher the percentage of patients with a resolved LVO on repeated imaging ($p<0.01$). CSC = comprehensive stroke center; CTA = computed tomography angiography; LVO = large vessel occlusion; NIHSS = National Institutes of Health Stroke Scale; PSC = primary stroke center.
